# (De)troubling transparency: artificial intelligence (AI) for clinical applications

**DOI:** 10.1136/medhum-2021-012318

**Published:** 2022-05-11

**Authors:** Peter David Winter, Annamaria Carusi

**Affiliations:** 1 School of Sociology, Politics and International Studies, University of Bristol, Bristol, UK; 2 Interchange Research, London, UK; 3 Department of Science and Technology Studies, University College London, London, London, UK

**Keywords:** Cardiology, Medical humanities, Social science, sociology

## Abstract

Artificial intelligence (AI) and machine learning (ML) techniques occupy a prominent role in medical research in terms of the innovation and development of new technologies. However, while many perceive AI as a technology of promise and hope—one that is allowing for more early and accurate diagnosis—the acceptance of AI and ML technologies in hospitals remains low. A major reason for this is the lack of transparency associated with these technologies, in particular epistemic transparency, which results in AI disturbing or troubling established knowledge practices in clinical contexts. In this article, we describe the development process of one AI application for a clinical setting. We show how epistemic transparency is negotiated and co-produced in close collaboration between AI developers and clinicians and biomedical scientists, forming the context in which AI is accepted as an epistemic operator. Drawing on qualitative research with collaborative researchers developing an AI technology for the early diagnosis of a rare respiratory disease (pulmonary hypertension/PH), this paper examines how including clinicians and clinical scientists in the collaborative practices of AI developers de-troubles transparency. Our research shows how de-troubling transparency occurs in three dimensions of AI development relating to PH: *querying of data sets*, *building software* and *training the model*. The close collaboration results in an AI application that is at once social and technological: it integrates and inscribes into the technology the knowledge processes of the different participants in its development. We suggest that it is a misnomer to call these applications ‘artificial’ intelligence, and that they would be better developed and implemented if they were reframed as forms of sociotechnical intelligence.

## Introduction

*‘The current system we have for diagnosing patients with rare disease is really not fit for purpose’*, says a respiratory disease clinician who is explaining the standard clinical practice of diagnosing pulmonary hypertension (PH). Pulmonary hypertension is a rare and fatal disease that, if left untreated, curtails life expectancy even further (Kiely *et al*. 2013). ‘*Survival is much better for the people who have been identified through a screening programme … you can run an AI algorithm that can potentially identify people who are at increased risk of the disease*.’ Healthcare professionals and patients alike are generally in agreement on the need to achieve earlier diagnosis for PH (Pulmonary Hypertension Association (PHA-UK) 2017) and highlights the general need for improving survival rates through earlier diagnosis of different diseases in clinical settings (eg, Blandin Knight *et al*. 2017; Ipsos MORI 2017; Naik 2021). In this regard, artificial intelligence (AI) algorithms in healthcare seem to offer hope of more effective diagnostic services, better quality of life, with unprecedented speed and accuracy (Harwich and Laycock 2018). This shift to AI illustrates healthcare professionals' orientation towards machine learning (ML) and deep learning (DL) approaches to bring about more early and more accurate diagnosis, believing that these new techniques will bring about a significant advance on traditional testing technologies (eg, biopsies, endoscopy, and medical images such as ultrasound and X-ray; Ahuja 2019).

There is much hype and positivity on the potential uses of AI in healthcare for diagnosis. However, only a few diagnostic AI applications are fully functional and routinely accepted in healthcare settings (Topol 2019). Even when claims are made regarding the high degree of diagnostic accuracy of AI in comparison to medical professionals (see, eg, Esteva *et al*. 2017; Rajpurkar *et al*. 2018), this does not mean that the AI application is accepted and implemented in actual healthcare settings (Cabitza, Campagner, and Balsano 2020; Nagendran *et al*. 2020; Sreedharan *et al*. 2020). Although AI is talked about as an already complete tool, ready for use in some domains, the development process of AI (like other technologies) plays a crucial role in laying the ground for the acceptance of the AI application (Elish 2018; Elish and Watkins 2020). In fact, the many claims regarding the positive potential of AI are counterbalanced by worries about its failings and implications, such as issues relevant to trust or mistrust (Asan, Bayrak, and Choudhury 2020; Jacobs et al. 2021; Lee and Rich 2021), accountability or responsibility (Elish 2018; Lysaght *et al*. 2019; Sendak *et al*. 2020; Sullivan and Schweikart 2019), bias (Challen *et al*. 2019; Cirillo *et al*. 2020; Gianfrancesco *et al*. 2018; Obermeyer *et al*. 2019; Tupasela and Di Nucci 2020), healthcare data set quality (Oakden-Rayner 2017, 2018; Laï, Brian, and Mamzer 2020; Scheek, Rezazade Mehrizi, and Ranschaert 2021), deskilling (Cabitza, Rasoini, and Gensini 2017; Floridi *et al*. 2018; Laï, Brian, and Mamzer 2020); job displacement (Recht and Bryan 2017; Strohm 2019); data privacy and security (Ipsos MORI 2017; Redmore 2019). Many of these issues involve a need for transparency or the lack thereof (Tonekaboni *et al*. 2019; Shortliffe and Sepúlveda 2018; Grote and Berens 2019; Harwich and Laycock 2018; Montani and Striani 2019).

Epistemic transparency, or rather, the transparency of knowledge, is a particular concern for healthcare professionals. As Shortliffe and Sepúlveda (2018, 2199) point out: ‘black boxes are unacceptable: A clinical decision support system requires transparency so that users can understand the basis for any advice or recommendations that are offered’. While AI has the potential to support and augment epistemic, or knowledge processes in the domains where it is implemented, it also threatens to disturb or disrupt them. The introduction of a tool that appears to do what humans do, without an understanding of how it does so, troubles transparency. This is not because everything about human knowledge and decision-making is transparent, but its opacities and transparencies are familiar and ordinary. AI troubles the familiar patterns of opacities and transparencies in high stakes domains such as medicine where life-changing decisions are routinely made. As this is behind much of the epistemic mistrust of AI technologies, our research aimed to get an insight into how issues of epistemic transparency are dealt with in the formative stages of AI development with a view to their integration and implementation in real-world clinical settings.

In this article, we describe the development process of one AI application for a clinical setting, where the very initial steps are being made before full validation (Winter and Carusi 2022). We show how epistemic transparency is negotiated and co-produced in the close collaboration between AI developers, clinicians and a biomedical scientist, forming the context in which AI is accepted as an epistemic operator. In the first section, we consider issues of transparency and visibility in AI applications for clinical use, against the background of discussions of these issues in software and algorithm studies. The form of transparency that we focus on is epistemic transparency, or an understanding of what software studies scholar Wardrip-Fruin refers to as ‘operational logics’ (2009, 13). We argue that rather than a process of understanding that comes into play after interactions with an already completed software application, the comprehension of ‘operational logics’ is brought into play during the process of development of the software, in the case of AI for medical settings. This means that there is a social dimension built into the very heart of these AI applications. In the second section, we describe three aspects of the development process of an AI application which has the potential for bringing about earlier diagnosis of a rare respiratory disease called pulmonary hypertension (PH), and for potential uptake in a clinical PH Referral Centre specialising in the disease. In the third section we discuss how these three aspects ‘de-trouble’1 transparency, laying the ground for the application to proceed to the next stages of development: that is validation and implementation.

We show how epistemic transparency is negotiated and co-produced in the close collaboration between AI developers, clinicians and a biomedical scientis, forming the context in which AI is accepted as an epistemic operator. In the first section, we consider issues of transparency and visibility in AI applications for clinical use, against the background of discussions of these issues in software and algorithm studies. The form of transparency that we focus on is epistemic transparency, or an understanding of what software studies scholar Wardrip-Fruin refers to as ‘operational logics’ (2009, 13). We argue that rather than a process of understanding that comes into play after interactions with an already completed software application, the comprehension of ‘operational logics’ is brought into play during the process of development of the software, in the case of AI for medical settings. This means that there is a social dimension built into the very heart of these AI applications. In the second section, we describe three aspects of the development process of an AI application which has the potential for bringing about earlier diagnosis of a rare respiratory disease called pulmonary hypertension (PH), and for potential uptake in a clinical referral unit specialising in the disease. In the third section we discuss how these three aspects ‘de-trouble’1 transparency, laying the ground for the application to proceed to the next stages of development: that is validation and implementation.

## Accepting AI as epistemic operator

The epistemic arena is much broader than knowledge, encompassing a broad array of attributes, capacities, actions and processes relating to the ways in which knowledge of different kinds can be acquired by different entities, individually or collectively (eg, Knorr Cetina 1999). The very terms ‘artificial *intelligence*’ and ‘machine *learning*’ position AI in the epistemic arena, while coupling these epistemic terms to ‘*artificial*’ and ‘*machine*’ marks them out as other to their usual human, or at least biological, instantiations. The discourse around AI frames the technology as carrying out or assisting with epistemic actions: AI ‘learns’, ‘identifies’, ‘classifies’, ‘predicts’, possibly even ‘decides’, ‘controls’. In this article, we take as our starting point this ordinary discourse that frames AI epistemically. This framing can be associated with both an acceptance of AI in the epistemic arena, and its exclusion from it. Our question is: *in what ways does AI become accepted as an epistemic operator?* The word ‘operator’ has many connotations: for example, sentential operators and logical operators are words (such as ‘and’, ‘or’, ‘not’) that make a difference to the truth value of sentences; they are also used in programming languages to execute functions. The word ‘operate’ also has more mechanistic or functional meanings, such as operations carried out by machines in order to perform a function that brings about a change in something. In this article we use the term ‘operator’ to indicate an active difference-maker, with affinities with Gilbert Simondon’s ‘theory of operations’ (Simondon 2017) and our use of the term ‘epistemic operator’ is meant broadly to indicate something that makes an epistemic difference. Our use of the term ‘epistemic operator’ is not, however, deeply theoretical for the purposes of this article.2 It lends itself to our purposes because conceptually it is neither human nor non-human, it can be used in many contexts (language, machines and any number of others). Many things make an epistemic difference, including human and non-human things, so AI is not at all unique in this regard. However, in the domain of healthcare and medical diagnosis, it is a newcomer, and has to be admitted and accepted as a difference maker to what is or can be known, or, that is, as an epistemic operator. The aim of this article is primarily to describe how AI is allowed to enter the field of epistemic operators in the first place, how it can even be considered in this light, by medical professionals, who are the traditional and established epistemic operators within clinics, working with tools, such as medical images and other technologies, which have long been admitted as epistemic operators in clinical contexts (see, eg, Joyce 2008). How AI came to be accepted as an epistemic operator is still not clearly understood. Our suggestion in this article is that this occurs through a process of *de-troubling transparency*—a process through which participants are able to recognise or inscribe their own processes into the AI. In doing so, they make it less alien and artificial and more familiar to themselves.

Although epistemically framed, AI is frequently perceived as a kind of ‘intelligence’ or ‘learning’ that is not fully understood, or possibly not understandable, by human beings. This however, is not unique to AI, and is well known in other forms of software (and other technologies). Software studies scholar Chun (2011, 18) writes about software as the ‘linking of rationality with mysticism, of knowability and unknown’ which is a ‘powerful fetish for programmers and users alike’. Even though software is a ‘paradoxical combination of invisibility and visibility’ (2011, 60), users gain a sense of mastery, confidence and competence through the direct manipulation made possible by interactive interfaces (Chun 2011, 63). Another way in which users can become more familiar with software is through what Wardrip-Fruin refers to as ‘operational logics’, which in the context of digital media and games, refers to structuring of the space of play (Wardrip-Fruin 2009, 71). Operational logics is a middle-level strategy of developers, which bridges between the technical logic of implementation (lower-level logics), and representations that are accessible to and understandable by users. This level of accessibility makes it possible for users to engage critically with the programme (Wardrip-Fruin 2009, 14). However, this form of accessibility comes into play only *after* the developers have completed their work. Rather than an *a posteriori* accessibility of already completed software programmes (or versions of programmes), we argue that there is a form of negotiation around visibility and invisibility, transparency and opacity. Such forms of negotiations are brought into the development process of AI for clinical applications, which is, like other forms of software development, deeply social in character. We take our point of departure from insights such as this from Seaver (2013, 10):

These algorithmic systems are not standalone little boxes, but massive, networked ones with hundreds of hands reaching into them, tweaking and tuning, swapping out parts and experimenting with new arrangements. If we care about the logic of these systems, we need to pay attention to more than the logic and control associated with singular algorithms. We need to examine the logic that guides the hand, picking certain algorithms rather than others, choosing particular representations of data, and translating ideas into code.

The key relationship in this process—the relationship on which depends the production of an AI system that is not merely notional or conceptual, but anchored in the clinical domain where it is to be used, is between AI developers (eg, statistical experts such as computer or data scientists and clinical experts (Elish 2018; Elish and Watkins 2020; Sendak *et al*. 2020; Winter and Carusi 2022). The relationship may involve only a few individuals, it may even be a one-to-one relationship for a significant part of the development if—as was the case for the AI applications we studied—they are pilots aiming for scaled-up application. Whatever the form, this relationship is essential for mobilising negotiations around transparency in the emerging AI system on the part of the communities in which each of the collaborators are embedded, and which they represent. Our study discusses three crucial stages when these relationships become intertwined with the technological system being developed, and inscribed within it: *querying data sets*, *building software* and *training the model*. In this process of intertwinement and inscription, the AI application that emerges is both social and technological. The ongoing de-troubling of transparency eases the way for the continued development of the application to full validation and acceptance in the clinical domain.

## AI for early diagnosis of PH

### The problem: diagnosing PH

PH is a rare and serious lung disease. According to the latest published figures, there were around 7000 people diagnosed with PH in 2017 (Pulmonary Hypertension Association (PHA-UK) 2017). The PH Association UK (Pulmonary Hypertension Association (PHA-UK) 2017) reported that 48% of patients would have waited over a year to be diagnosed after first experiencing symptoms, and about 10% of patients took over 3 years to be diagnosed from onset of symptoms. This has an effect on life expectancy and quality of life. It is generally agreed that getting an early diagnosis is crucial for improving the quality of life of those with the disease, and increasing life expectancy (Pulmonary Hypertension Association (PHA-UK) 2017).3 However, obtaining early diagnosis is the major challenge of this disease area. Improvements in the precision of tests for the disease have not been correlated with improvements in the rapidity of diagnosis.

As one respiratory clinician explained: ‘*what we’ve seen over the last twenty years are: one, a lot more patients being diagnosed and two a lot of patients living an awful lot longer; but despite that the time from initial symptoms to diagnosis for some forms of pulmonary hypertension has remained unchanged’*. The group of clinicians and biomedical scientists who participated in our study have a strong prior record of technological innovation and early adoption (Godin 2019). Approached by a leading bioinformatics company working in the broad area of data applications for healthcare, they were keen to take up the challenge. The process of iterative development through close collaboration could be understood as one where users are configured for technologies, rather than technologies for users (eg, Woolgar 2014); or it could be understood as an instance of co-shaping, where technologies and users co-evolve (eg, Suchman 1987). Our position in this article is closer to the second. However, most importantly, we aim to show how these processes of collaborative development problematise the artificiality of AI, and instead show the extent to which it is human and social (Suchman 2006).

## Methods

Research for this article was conducted through fieldwork at a UK Pulmonary Hypertension Referral Centre at a major National Health Service (NHS) Teaching Hospital, where three AI applications were being developed simultaneously. These applications were all at early stages of development, and our research methods were aimed at eliciting details about the process of development as described by those involved in it. The study of the screening algorithm—which is the focus of this article—took place against the background of the broader study of all three applications. The broader study documenting the development of all three applications is described in (Winter and Carusi 2022). Fieldwork included interviews and observations of the interactions and discussions, of ideas, scientific criteria and concepts that shape transparency in AI development. The focus of this article is specifically on analysing professional accounts of the development of the screening algorithm for idiopathic pulmonary arterial hypertension (IPAH). This particular application made use of Hospital Episode Statistics (HES) data collected by the NHS as well as locally generated data sets collected by the PH Referral Centre or their academic counterparts. The term ‘algorithm’ is used throughout because it was the term that all of the participants of our study used to describe the AI that they were developing. Three interviews focused on the development of the screening algorithm. These were conducted face to face in workplace offices. Data were collected between 17 May 2019 and 22 October 2019. Recordings were transcribed and uploaded to NVIVO V.12 to help manage, code and analyse themes that emerged from the transcripts. Using a ‘thematic analysis’ framework (Braun and Clarke 2006), we identified recurring and contrasting motifs related to transparency as talked about by professionals. Through analysis of the themes, we clustered them into the main themes discussed in the paper: issues of transparency were ubiquitous, and the three activities we have described were the three main ways that participants talked about the process of collaboration.

In addition to interviews, we conducted fieldwork observations of two types of weekly multidisciplinary team (MDT) meetings: on the PH ward and in the radiology department in which decisions concerning diagnosis and treatment of patients are made. Observations were recorded as fieldnote data and served three purposes: first, observations provided valuable opportunities for learning about the disease (eg, terminology) and the diagnostic process, second, observations helped build rapport between participants and researchers, and third observations supplied information for events or situations that informed the development of appropriate interview questions at a later date. Therefore, observational data augmented the interviews and thus served as a useful purpose. In terms of limitations, this qualitative study was based on a relatively small group of participants (n=3) involved in the development of the screening algorithm for the PH Referral Centre. Such a limited number of interviews will affect the generalisability of our findings. While it is a small study, it has many points of convergence with research conducted by Elish and Watkins (2020) by producing some important insights on healthcare AI development, where to date there has been limited work in this area. This calls for ongoing research into how interpersonal, social relations between people with different disciplines and expertise, purposes and concerns, play a role in the development and acceptance of AI in clinical settings.

## Three processes of development: querying data sets, building software and training the model

In this section we describe three interrelated dimensions of developing AI for IPAH as they emerged from interviews. A consideration of these three dimensions of AI development brings into focus the challenges of developing the screening algorithm for optimising IPAH diagnosis. This process involves: *querying data sets*, *building software* and *training the learning model*. We discuss the ways in which these three tasks are talked about and how the practical work of clinicians working to develop the algorithm has the effect of mitigating issues of transparency associated with AI, in effect, making this less troublesome.

### Querying data sets: ‘That’s where we get the ground truth label from’

AI algorithms rely on data sets for training and testing the algorithm’s capacity to learn. This means that the querying of data sets and process of checking the quality of datasets by clinical experts are the crucial first steps of any AI development (Laï, Brian, and Mamzer 2020; Oakden-Rayner 2017, 2018; Scheek, Rezazade Mehrizi, and Ranschaert 2021; Sendak *et al*. 2020). In our study, the querying of clinical data sets was a major recurring theme throughout our conversations. The data scientist, clinician and biomedical scientist emphasised the need for querying data sets in order to assess the quality of their data source and curation. The quality and curation of data sets were seen to provide the foundation on which everything else would rest, highlighting the ‘need for high-quality health data’ in the development of AI tools (Laï, Brian, and Mamzer 2020, 11). Only if a data set was curated in terms of precise labelling, codes or complete annotations, would collaborators be confident that it was accurate enough to qualify as data they can act on. They argued that any claims about the performance of the AI would depend on whether the labels *‘pulled’* from the data set came from clinically curated data sets. In our study, collaborators’ accounts of querying the quality of data sets resonates with contemporary concerns that data sets bring with them new and important uncertainties in the form of biases and errors, and as a result, new diagnostic challenges that require urgent attention and analysis (eg, Challen *et al*. 2019; Cirillo *et al*. 2020; Obermeyer *et al*. 2019). Importantly for this study, we want to stress that, through the querying of data sets, the term ‘bias’ was used to refer to the ways in which clinical diagnostic processes may lead to biases. It rarely referred to social, racial or economic biases that may exist in the data set, although the full range of PH diseases is associated with gender, socioeconomic and racial profiles (Talwar *et al.* 2016; Yang *et al*. 2018). However, because the clinical experts in our study were using their own clinically curated data set which reflected the demographics of their every day clinical experience and practice—this kind of bias was not what they were querying. A case can be made that they should be, but this is not the focus of this article.

A primary strategy of the team developing the screening algorithm for IPAH was to engage the clinicians early on in the particular task of querying the quality of data sets and determining whether labels or codes were accurate or precise. For example, one clinical expert (Participant 1) spent time collecting and looking at data sets from existing clinical repositories that were relevant to the diagnosis of IPAH. These included both external (eg, HES, Electronic Medical Records from *‘NHS Digital’*) and internal data sets based on patients that referred to the centre (*‘and we looked at our own patient population’*)—a secondary use of existing data that has come to be known as ‘repurposing’ for purposes ranging from research to quality improvement (Bonde, Bossen, and Danholt 2019). For the data scientist, clinician and biomedical scientist, it was important that the kinds of labels with different diagnostic characteristics were queried and confirmed to be IPAH (*‘true positive’*) and not to be misleading, or hopelessly vague. Here, the clinician collected a local research data set for specialist information on IPAH, asked for external data sets, performed a level of cleaning (eg, checking how other clinicians used International Classification of Diseases, Tenth Revision (ICD-10)4 codes to classify patients with IPAH/non-IPAH/PH) with assistance from another colleague, and recorded according to whether the other colleague agreed or disagreed. This clinician spent much time studying and interpreting this information, a process that was enhanced by linking these data sets together and cross-referencing ICD-10 codes. Hence, according to the data scientist working with this clinician it helped *‘build in trust to the dataset from the beginning’* (Participant 2).

Even though there is broad convergence on the application of diagnostic codes across Trusts, there can still be a degree of variation. Coding sits within a complex clinical, management and financial model, and achieving national consistency may be challenging, depending on local practices and pressures (Van Baalen and Carusi 2019). Therefore, querying data sets involves more than merely the observation of diagnostic codes and information about tests, but also rebuilding and sometimes guessing at the local context of external data sets. As a result, this often poses problems of transparency—a problem that is explicitly highlighted by the biomedical scientist (Participant 3): *‘you know it’s not the most standardised dataset, with coding often defined by local practice […] but it is what it is, and as long as you use it with your eyes open, and know the limitations of it then that’s the important part of using it’*. Querying and recoding creates a transparency adequate for the work at hand. (Re)constructing the codes for the data sets, including the clinical data, measurements, diagnostics and follow-up tests that go into them is a major research focus in AI development, and there is great concern to guarantee ‘carefully curated’ data sets (Sendak et al. 2020, 6)—something frequently expressed by the experts in our study.

This cautionary note is supported by evidence that it is common for those accessing outside or external data sets to have concerns with the definition of labels and quality of diagnostic data (Laï, Brian, and Mamzer 2020), and often includes tasks of recoding or relabelling distributed among healthcare professionals (Oakden-Rayner 2018; Oakden-Rayner 2017; Rajpurkar *et al.* 2018). Importantly, the biomedical scientist who works on the screening algorithm, praises the MDT for being able to convert a *‘diagnosis’* into a *‘ground truth’*:

Our ground truth of diagnosis is not based on one individual. We use diagnoses derived from MDT meetings so you’ve got a consensus decision. But I think it is an important point. We should definitely make sure we know how external data have been curated. In biology we often replicate findings using alternative methods and in health data science) we should make sure that we are (where possible) not just relying on one person’s point of view when we’re publishing. (Participant 3, Biomedical Scientist)

The scientist suggests that MDT meetings provide the ground truth. It is exactly what the data scientist means when they say: *‘that’s where we get the ground truth label from’* (ie, from the local data set) and specialists figure out what characterises each patient and where diagnoses are labelled with the correct categories (eg, distinguishing PH from non-PH or a specific type of PH). MDT meetings are opportunities to provide a forum where participants converge on similar decision-making processes, and play an important role in the introduction of any new technology (Van Baalen *et al*. 2017). The ICD-10 coding practice, built gradually over time and cross referenced in multiple ways had set up a data set from locally collected clinical data and reflected the spectrum of PH encountered at the PH Referral Centre between 2001 and 2010 (Bergemann *et al*. 2018; Hurdman *et al*. 2012). This highlights the shared development of a key data set and the internal familiarity of specialist experts involved in assessing the quality of the codes (ie, ICD-10 codes) for PH. Doing so created a transparency adequate for querying, comparing and cleaning codes.

There is an assumed transparency about ‘local’ data sets: collaborators are aware that the local data set (that contains PH diagnoses) goes through MDT meetings whose ICD-10 codes fit and flow in intensely monitored group discussions through thoroughness and accuracy. This is the basis for the data scientist’s evaluation of the MDT meeting where the codes are *‘confirmed to be Idiopathic Pulmonary Arterial Hypertension, so that’s where we get the ground truth label from*’. Yet, despite their seeming confidence of the internal PH data set, a close examination of data drawn from external sources reveals their uncertainty over how other clinicians at other hospital Trusts or PH services used ICD-10 codes to classify PH. External data sets, where the coding is not ‘first-hand’ raise questions about how those diagnostic codes were arrived at.

The crucial point, however, is that the reason why the codes are considered ‘*standardised’* or *‘curated’* in this context is because of MDT meetings. The MDTs are the prime generator of the internal data set that is being used alongside the external HES data set to train the screening algorithm. This was evidenced by the reference to MDTs in the research article the group published in the building of the local data set: ‘diagnostic classification was by standard criteria following multidisciplinary assessment by experienced pulmonary vascular physicians and radiologists’ (Hurdman *et al*. 2012, 946). Participation in this labelling/relabelling (or ‘cleaning’) of data gives the clinical collaborators an understanding of the data on which the AI will operate, even if they don’t have a direct understanding of how the AI works.

This collaboration between AI developers and PH experts builds transparency into the algorithm through using a data set that is familiar to the clinician collaborators, where they have a good overview of its provenance and features. Hence, the data scientist repeats the assertion that the thoroughness, the accuracy, the diligence and the fastidiousness of the internal data set is *‘where we get the ground truth label from’* (Participant 2). It is after all the ground which forms the basis for the research and structures the way the algorithm learns. In addition, the attempt to query data sets—to repurpose, critique relevance and usability, and to distinguish good data from bad data—allows us to see data as something that is not discipline-bound but as something that seems to constitute their own ‘data community’, in which a new community of practice is formed (Gregory *et al*. 2020). If this is the case, then we can see that having clean data sets and agreed on codes or labels lays the groundwork for the software that the clinicians use.

### Building the software: ‘There’s been so much work in the community of making excellent packages for things like gradient boosting trees’

In our study, the data scientist collaborates closely with the clinician and the biomedical scientist in the development of ML software. We found that an important role was played by the choice of software. A ‘baseline’ off-the-shelf software program was chosen, and progressively modified to suit the purposes set by the collaboration. The choice of software and ongoing refining of codes or variables seems to take advantage of the clinician’s experience of diagnosing the disease (aspects of which are likely to be familiar to those working in the PH Referral Centre). This raises the question as to whether the clinicians who get involved in these kinds of collaborations are already more attuned to the predictions of model outputs.

An important insight of the collaboration during this stage of the development process was that common statistical software was being used in the laboratory. The data scientist targeted a software application that was popular among members of the computer science community, as a first step to establishing a common framework for the collaboration: this had the benefit of facilitating the production of explainable, interpretable and understandable outputs. With a software program already held in common, the interpretation of the outputs of the ML algorithm becomes much easier. This allowed the collaboration to proceed through methods already established within the scientific community. On their part, the data scientist was more convinced of their outputs from algorithms that are developed as *‘off the shelf’* software readily available for potential collaborators. Familiarity with the software conventions in the field (such as *‘XG Boost’*) and including the clinician and biomedical scientist in these conversations are built into the further development of the program for the specific purposes at hand. This takes the form of iterative refininig or tinkering of the software program chosen as the baseline. It is part of a practice in which the clinician and biomedical scientist learn to emulate their collaborators’ forms of knowledge, and also allows the insights from the data scientist’s practices to be taken up by the clinicians. The quote below, taken from the biomedical scientist, highlights the use of a standard software package that pervades popular perceptions about ML approaches, and what software packages are being used in laboratories:

At this stage everything has been machine learning based approaches. So, using machine learning tools, that are standard ‘off the shelf’ tools. In our case we are using XG Boost which is a popular and powerful package. (Participant 3, Biomedical Scientist)

Similarly, the data scientist working on the screening algorithm values the XG Boost as a software that provides a framework for *‘gradient boosting’*, which is *‘probably one of the most well used models at the moment in this case’*. In their interview, the data scientist revealed the software’s advantage in facilitating the collaboration of *‘trees’*, to help make errors transparent, and to mine for connections with classification power. In their words:

So *mainly* this idea that the trees work collaboratively. So, if you build the first tree then the next tree focuses on the error of the first. So, they try to really focus on those hard to classify observations which makes them- in *some* cases, not all, but more powerful than the random forest. They’re also, from a computational point of view, *far far* faster, which is *counter intuitive* because for me […] I think of parallel processors like I’m building parallel trees of a random forest. I think that must be faster than something doing iterative. But because they’re *so popular* in all of the machine learning competitions there’s been so much push from the community to make very clever implementations of them, they’re very very efficient. So there’s been so much work in the community of making *excellent* packages for things like gradient boosting trees; a little more than random forest. (Participant 2, data scientist)

The data scientist further claims that the gradient boosting trees differ from the more primitive random forests by virtue of being *‘more powerful’* and *‘from a computational point of view, far far faster’* to arrive at a classification but also suited for extremely challenging and difficult tasks. The data scientist also illustrates the software as a popular choice among computer scientists in the ML community, further signalling a common framework they would like to embed their work in, but also, in part, bowing to the pressure to use the software in the context of heightened competition (*‘there’s been so much push from the community to make very clever implementations of them’*).

Alongside the steps being taken to build the software, our participants also highlighted how the initial architecture of the screening algorithm is based on supervised learning. Supervised learning was constituted by a human’s process of code and variable selection, wherein the learning model for the screening algorithm was based on the clinician’s and biomedical scientist’s role in the selection of these data. In addition to this supervised component, the biomedical scientist provided useful insights into how the screening algorithm can be improved by a *‘biomarker algorithm’* that was guided by *‘unsupervised learning’*. It was anticipated that this unsupervised component would be included in the screening algorithm to provide more specific data on the mechanics of the disease. The ‘biomarker’ algorithm therefore pointed towards a more exploratory orientation to classification, indicating a possible route for unsupervised learning. For example, they described the potential usefulness of unsupervised learning in software development for the biomarker algorithm for extracting and classifying vast amounts of data from *‘lots of different datasets’* (eg, *‘transcriptomic’*, *‘metabolomic’*, *‘proteomic’*, *‘genomic’*, *‘epigenetic’*). This capacity of the biomarker algorithm transcends organisational boundaries for experimental protocols and discovery (*‘how you link all that together into known pathways, biological pathways, and networks of genes and so on’*). While they talked about supervised in terms of a human selecting *a priori* labels of data from training data sets, they talked about unsupervised learning as occurring when an algorithm establishes labels or classifications itself based on any intrinsic regularities between the data (sets).

However, the more humans are involved in refining, for example, the grouping and clustering of data, the more likely that the process will be thought of as supervised learning. The repeated refinement of the algorithm (*‘each time someone goes through it’* as the biomedical scientist put it) brings into play the human and social processes and real-world settings of clinical researchers. The supervised approach brings algorithm development closer to the clinician. Supervision of the screening algorithm provides opportunities for the biomedical scientis and clinicia to participate in its development, and this contributes to building confidence in the design of the software. In the process of iteratively improving the software they become aware of any errors it makes, as in the case of the ‘gradient boosting trees’ (*‘you build the first tree then you focus on the error and you build the next tree focus on the error, and you build the next tree, so you’re trying to—all the time you’re trying to improve’*: Participant 2, (data scientist). This point, we argue, ultimately influences the technology’s adoption and conditions for transparency in clinical practice.

A third practice of the collaborative approach to software development used in the project was the selection of other variables besides the diagnostic codes to be applied to the data. Together with the codes for classifying the data set, these further variables are a bridge between the data and the software on one hand, and between the software and the diagnostic practices on the other. The assessment of the software is based on how it applies the diagnostic codes because the entire point of this use of AI is to arrive at earlier diagnosis. In the case of the screening algorithm, industry researchers from the bioinformatics company conducted interviews with Participant 1, a clinical expert in the diagnosis and treatment of PH, concerning the variables or other criteria used in their practice:

As physicians there was a degree of involvement in terms of what we *thought* were important variables that may identify or exclude individuals with PH. So, in the development of this algorithm we were interviewed by people from a bioinformatics company, just asked quite a lot of questions about the disease, also asked about other *diseases* that may present in a similar way to idiopathic disease. So, actually when we’re building up the algorithm, it wasn’t just a machine in isolation just looking at patterns of behaviour. There was interaction with people who were expert in the management of the condition, *but* I think what was *also* quite important was we were asked a lot of questions about the disease but we weren't necessarily given specific feedback as to *why* we were being asked the questions. So, I guess what the guys wanted to do was get information from ourselves without necessarily overly *biasing* the system. So, I think that’s the sort of difficulty. If you’ve got a, you know, an AI approach then I guess one of the difficulties is that if it’s just the *machine* itself, there might be important things it doesn’t potentially recognise. But if it’s *individuals* then you may exclude lots of parameters that actually may be important that aren’t recognised as being important. So, I guess it was a bit of a half-way house. (Participant 1, clinician)

The clinician’s own description of their interview with the bioinformatics company shows how the clinician’s experience was integral in selecting the most appropriate variables or criteria relevant to the diagnosis of PH. The clinician highlights the initial work of *‘what we thought were important variables that may identify or exclude individuals with PH’* from the diagnostic coding of the disease. Alongside this, it appears the developers also wanted to know about other common respiratory diseases (such as *‘asthma’*, *‘COPD’*) and symptoms (*‘breathlessness and fatigue’*) that would often be coded in error or misdiagnosed (*‘also asked about other diseases that may present in a similar way to idiopathic disease’*). This is because the rarer the disease, the greater the likelihood of misdiagnosis (Kiely *et al.* 2013). While the clinician’s experience is solicited, the clinician remarks that *‘we weren’t given specific feedback as to why we were being asked the questions’*). Hyperaware of the possibilities of bias emanating from clinicians themselves, the clinician takes this as a reassuring sign that the industry collaborators are also aware of this (*‘I guess what the guys wanted to do was get information from ourselves without necessarily overly biasing the system’*), and are taking steps to address it that are not only technical (*‘not quite a machine in isolation looking at patterns of behaviour’*), but also show awareness of human and social sources of bias (*‘there was interaction with people who were expert …’*).

### Training the model: ‘What can I solve myself by looking at the data and then what can I raise to the clinician to say “this looks kind of strange?”’

Having been involved in querying the data sets and in some of the steps for building the software, clinical collaborators are further integrated in the development process. Training and discarding or refining the model prepares for the validation of the software, both internally and in the context of the real-world clinic. Training occurs through several iterative steps, with assessments of the outputs at each step. This process ensures that the algorithm does not contain unnecessary information, that all data considered relevant are included and that all outputs are in fact comparable with relevantly similar clinical judgements. This was the case, for example, with the joint effort of collaborators (ie, data scientist and clinician) working together to make sense of the training outputs in the development of the screening algorithm. In the context of an ML laboratory, with retrospective processing around patient data from multiple data sets, the data scientist needed to draw on their clinical collaborator as a source of information and insight into training outputs that seemed strange and suspicious:

So what can I solve myself by looking at the data and then what can I raise to the clinician to say ‘this looks kind of strange?’ So yeah and I think that’s what’s hugely valuable is if you can have a clinical expert to be part of the development procedure. I found that to be just priceless because he and the team saw all of the things that we did, they saw when we were worried, they saw when we were like ‘no this actually looks okay now’ and I think you can’t put a price on the value of that in growing the trust. (Participant 2, data scientist)

The remark that *‘this looks kind of strange?’* when looking at the initial test results of the model implies that the output was not what the data scientist expected. The data scientist’s confusion is clearly articulated within the quote, and in effect requires the clinician’s presence in making sense of the output when the output leaves them unsure. The data scientist feels that it is *‘hugely valuable’* if a clinical expert is part of this process. It is a process that guides them towards the preferred interpretation and helps clinicians familiar with the data to participate in a type of ‘relational looking’ (De Rijcke *et al*. 2014, 147)—an emerging kind of looking through which the model is seen in relation to the real-world clinic. Of test results that were thought to be suspicious, the data scientist noted:

If it’s very good that’s usually an alarm bell. If you build your first model and it’s really good I'm generally filled with *doom*. We’re like: *‘Oh! What is happening here? It shouldn’t be that good, this is a really hard problem!’* and that’s what we did have in one of these scenarios where we ended up having a really good model and we went to the Hospital, we kind of went down every two to 3 weeks, maybe every 3 weeks, and we said ‘the model’s really good, we’re really worried!’ and we got the clinician and the biomedical scientist to sit down and look at all of the outputs and we never showed them what the result was, we never wanted them to see the performance metric because I knew they would only be devastated in the future when I give them a more realistic model so we never told them what the accuracy was but we just said ‘we don’t trust it, I’m *not* even going to tell you that, but please look at all the outputs I’m getting and help me understand what’s happening!’ (Participant 2, data scientist)

We can also see the ‘too good to be true’ suspicion from the perspective of the clinician:

And so when we were given data and feedback from the performance of the algorithm then we would review the data and think about whether it, you know, this seemed reasonable and would we want to make any refinements? So, I’ll give you an example: when you come into a hospital and you’re *coded* (Pause) (you know for the diagnosis), a lot of people with idiopathic disease don’t get a diagnosis of PPH [primary pulmonary hypertension], which is how it’s coded using ICD-10 codes, (a lot of people) aren’t given a diagnosis of PPH. They might be given lots of *other* diagnoses, some of which are erroneous. But when the patient is then subsequently diagnosed with pulmonary hypertension, often just before referral, you know you can identify on *coding* - a coding number ‘one’ for primary pulmonary hypertension. Now, if you had an *algorithm* that uses the diagnostic code including PPH then it’s very very good at identifying patients with pulmonary hypertension, not surprisingly. (Participant 1, clinician)

Although the algorithm is meant to be helping the clinician arrive at a confirmed PPH diagnosis more quickly, suspicion arises when the outputs of the algorithm are too accurate. Looking for a reason for this, a query will be raised concerning the data that the algorithm was trained on. From their clinical experience of how different diagnostic codes, and sometimes no codes, can be used prior to referral—that is, prior to a confirmed diagnosis of PPH—the clinician knows that the input into the algorithm should not include the codings that the clinicians are already sure of. If the algorithm is trained on the data that includes the PPH diagnosis, its results will be too good (*‘not surprisingly’*). The high level of accuracy of the result will elicit a suspicion of circularity: that is, the algorithm’s outputs are already presumed in its input. To clarify this point, the clinician recalls a *‘nice study’* published in the journal *Artificial Intelligence* about a ML algorithm that *‘read scans to see if they (the patients) were likely to have PH or not’*, however, *‘what the machine actually ended up reading was […] I think on the bottom was a certain type of cancer hospital’*. The clinician’s point highlights how it may be unknown which information algorithms actually pick up (in this case the label or name of the cancer hospital instead of disease/non-diseased anatomy): *‘it was actually using the information that was there and creating the algorithm based on that rather than looking at the images’*. What the algorithm was doing here was automatically learning the shortest or easiest route for the navigation of the image and in effect building a relatively simple model with a small number of features and their interaction. This is one reason why AI developers wish to continue reviewing the input data (as well as output data) in order to check whether the algorithm does not have this type of circularity. This potential for circularity requires evaluating and refining the ICD-10 codes.

The potential for circularity is by definition related to a challenge that overshadows the whole process of developing the learning algorithm, and that is building a *‘trivial’* learning model from the experimental algorithm. For instance, the data scientist demonstrated that algorithms adapt their learning to find the easiest path and simplest rule to predict the classes of all the training data:

What you really want to do is not build a *trivial* ML model because machine learning is really good at finding the *easiest* way to separate lots of data and if I have everyone in my dataset that’s really healthy, hopefully, then it’s really trivial to say: ‘here are quite ill people and here are people who are fine’, tell me how they are different? I mean it just doesn’t make sense, right? And it’s not something that replicates what’s happening to the clinician’s clinic, right? I don’t walk into the clinician and say: ‘do I have IPAH?’, right?’ (Participant 2, data scientist)

Such simple predictions based only on the presence or absence of a rare disease fail to capture actual practice, where there is little or no information about the disease or imprecision from clinical notes about time of diagnosis or about coding practices. Technically, both circularity and triviality arise from overfitting. Overfitting occurs when a model fits its training data very well, but fails to generalise to other data sets (Domingos 2015). A major concern is ensuring that the training data set contains the right data points. In the case the clinician and data scientist are describing, this means ensuring that data with the right amount of uncertainty is included in the training set. One of the points mentioned by the clinician is that it makes a difference *when* codes are applied. Variables that are clearly relevant in the context of the ‘too good to be true’ model are those that relate to time. So, in the data set for the screening algorithm, three different forms of data are crucial: one of them is the *‘ICD-10 codes’* (the diagnostic codes), and the other two are temporal: in the selection of *‘index dates’* (the date from which the patient’s history begins for the purposes of training the learning algorithm) and the *‘lookback dates’* (how far back in the history to go).

However, this emerges over the course of their discussions and collaboration. Identifying the correct index dates and lookback dates, and interpreting the use of the ICD-10 codes relies on the expertise of the clinicians, and in particular their tacit knowledge of the domain. In eliciting their knowledge for building the data sets and for building the software, there is much that is not made explicit, because neither the data scientist nor the clinician know in advance what will be relevant. This knowledge may be tacit also in that it remains in the background until invoked by something in the development process, such as ‘suspicious looking’ outputs of the learning model. At this point too, the clinicians’ knowledge of the clinical context is brought to bear and made explicit: knowledge about the temporal progress of patients’ journeys through the health system as they seek a diagnosis, about the progress of the disease, and about the way that diagnostic codes are applied. In addition, the data scientist points to the role of the active participation of the clinician in establishing trust: (*‘you can’t put a price on the value of that in growing the trust’*).

Choosing the dates between which data should fall is pivotal in negotiating a model that is a pragmatic compromise between realism and triviality. The data scientist explains:

So, what does an index date mean? So, if I had information about someone for ten years and they’ve had IPAH for 6 months then what part of their patient history do I look at? Do I look at many years ago and make it very hard and unrealistic? But also I don’t want to look at the day before they got diagnosed. Because if I use information the day before they got diagnosed, someone like the clinician *probably* already has a suspicion or has maybe already made up his mind but he’s *waiting* for a final confirmatory test. So, therefore if I say to the clinician: ‘oh, clinician, I could have told you yesterday that person with IPAH [quietly: well it can’t be yesterday] so how do I make it clinically valuable to them?’ (Participant 2, data scientist)

To combat the problem of overfitting in the trained model the data scientist wants to refine the index date because the algorithm has a tendency to take care of the trivial associations. When a suitable ‘index’ date and ‘look back’ date (*‘how far back we go’*) are negotiated and collaborators are confident in the pragmatic soundness of their variables, they transition to ‘validation’. Only then do most of the uncertainties associated with training the model emerge, often with further challenges. This is a crucial aspect of the refining and to-and-fro dialogue that occurs between AI developers and clinical collaborators. There is an interplay between uncertainties and expert, often tacit knowledge, in the development of the learning model.

We have already spoken about the ongoing ‘refinement’ of variables or criteria that play out in the continual movement between ‘data’ and ‘outputs’ in software building (see Winter and Carusi 2022). These refining or tinkering practices open up a space for building the software and training it together, and contextualises the codes so as to give traction and meaning to diagnostic codes and variables selected for the model. The outputs of the software are most interpretable to those who have participated in building the software—this interpretability is crucial for the assessment of the outputs of the software.

## Discussion

One of the most important challenges to introducing AI into clinical contexts and including it in epistemic tasks is that of transparency: that is, the clinicians’ or other healthcare professionals’ lack of knowledge or understanding of how the outcomes of the AI software program have been arrived at. Even though clinicians are used to working in contexts of relative black-boxing of how judgements are made, in the case of their colleagues, they rest on assumptions of similarity of human reasoning; and in the case of the many other technologies they already rely on, the mediation by other expert colleagues plays an important role. AI or ML are both initially framed as being a departure from the norm, because they are cast as both having qualities normally associated with humans (intelligence and the capacity to learn), but with a distinctly non-human component (artificial and machine). We found that no matter how they are initially framed, within a context of producing an AI or ML application, there is quite a lot of discussion of what the terms actually mean. The troubling of transparency that AI applications can result in has to be mitigated if they are to be trusted sufficiently and used in clinical contexts, rather than existing only on the pages of journal publications. We have shown how the central collaboration between data scientist and (in our case) clinician and biomedical scientist builds up familiarity with the application, and brings in a level of transparency at least sufficient for the interpretability of the outcomes of the programme for clinicians. This is similar to the comprehension of the ‘operational logics’ (Wardrip-Fruin 2009) of the programme by its users, except that in this case, potential users are built into the programme from the outset. We suggest that there are three key stages where this is achieved: through involving clinician users in *querying data sets*, and thereby ensuring that the data on which the algorithm is trained is trusted by the clinicians from the outset; through including them in *the building of the software*, in particular by capitalising on the familiarity that clinicians already have with particular software packages and processes, and involving them in specifying the codes and variables that bridge between the data, the software and the contexts of use; and through the ongoing refining and iterations of *training the model*, which enable data scientists and clinicians to come together to an agreed set of criteria to assess the model, which are interpretable by all concerned. These are all ways of de-troubling transparency for users, at least to a sufficient degree for them to be willing to take the development of the application to its further stages and finally validation (which we deal with in a separate article). It is in this process, we claim, that AI applications become accepted as epistemic operators in clinical domains. However, as epistemic operators, they are by no means purely artificial, as they bear the traces of the many interpersonal and social interactions that have shaped them, and inscribed into them the knowledge processes of developers. We believe that further research is warranted of the many different ways through which this occurs. A framing of these technologies as a sociotechnical form of intelligence would attract more attention to these dimensions of development and implementation, and hopefully, produce better attuned epistemic operators.

## Conclusion

Many claims are made concerning healthcare AI. These range from quite modest claims regarding the usefulness of AI technologies in assisting healthcare professionals, to the rather more immodest claims of how AI will revolutionise healthcare. Yet, the acceptance of these technologies being used for diagnostic purposes in real clinical contexts remains low (Topol 2019). This discrepancy indicates that there is a gap between the claims made about AI in healthcare, and its actual use. In this article, we follow the development of AI for clinical settings in order to gain insights in what makes AI more useable. Here, we have provided insight into the way clinical experts are embedded into three different modes of social collaboration in the development process for the earlier diagnosis of a rare disease. These collaborations include tasks such as querying data sets, building software and training the model. Through collaborating on these tasks, the epistemic processes of people in their context of use become inscribed into the algorithm. This has the effect of making the AI application less opaque to the clinicians, and therefore more acceptable to them, at least to the extent that the applications are sufficiently endorsed to go forward in the validation process. On the developer side, the applications become less abstract, and more anchored in the context of use, which makes them interpretable in that context. We claim that collaboration and interaction in these three stages of the development process allow clinical experts to accept the AI application as a potential epistemic operator. This highlights the extent to which AI is not ‘purely’ technological, since the form that it takes is so thoroughly intertwined with the practices and experience of people in its context of production and use—whatever those are. The implication of our study is that the ‘artificial’ is a misnomer for this form of profoundly sociotechnical intelligence, and that framing it as such would bring more realism to its development and implementation.

## Data Availability

Data are available upon reasonable request.

## Notes

1. In peer review, one anonymous reviewer suggested that our term ‘de-troubling’ was an implicit reference to Haraway (2016, 1)’s concept of ‘staying with the trouble’ which describes how knowledge production can generate productive and also challenging frictions, a dichotomy that allows those to ‘be truly present’ in the ‘myriad unfinished configurations’. While it was not our intention to draw on Haraway for the purposes of this article, the collaborators’ process of adapting to the challenges of AI development (such as bias or imprecise labels in external data sets) can be seen as an example of ‘staying with the trouble’ and provides a point of connection between our study and Haraway’s. This is an important consideration for our study since collaborators often highlighted these issues as part and parcel of the early development stage and necessary for development to proceed. At this early developmental stage, the algorithms just needed to be adequate for the work at hand (ie, as a proof of concept in the laboratory) subject to ongoing refinement (see Winter and Carusi 2022). This calls attention to a direction for further work which might study how his process of ‘staying with the trouble’ proceeds, and when (or if) it ever stops.

2. An extended theoretical treatment of AI on Simondonian lines is not possible within this article. However, the affinities between AI and other technologies such as medical images would certainly lend itself to this theoretical framework. For a theoretical treatment of images as operators in Simondon’s sense see Hoel (2020).

3. In our fieldwork, and in fieldwork for a prior project involving the same community, we heard only one dissenting voice querying the money poured into funding new technologies, rather than treatments such as physiotherapy. Even though in the minority, further research on these dissenting voices in the clinic would be worthwhile.

4. International Classification of Diseases, Tenth Revision (ICD-10) is a globally used diagnostic classification tool which contains codes for diseases, signs and symptoms, abnormal findings, complaints, social circumstances, and external causes of injury or diseases. This article is squarely focused on the use of ICD-10 codes to identify potential patients with pulmonary hypertension (specifically IPAH). See Bergemann *et al*. (2018) for more information.

5. Primary pulmonary hypertension (PPH) was defined in 1973 by the World Health Organisation (WHO) as being characterised by vasculopathy of the pulmonary arteries. However, better understanding of the disease led to its reclassification as 'idiopathic pulmonary arterial hypertension' (IPAH) (Kiely et al. 2013, 1). Patients who have IPAH may still receive the diagnosis code of PPH because it was previously coded as PPH using ICD-10 codes.
